# p53-Independent Cell Cycle and Erythroid Differentiation Defects in Murine Embryonic Stem Cells Haploinsufficient for Diamond Blackfan Anemia-Proteins: RPS19 versus RPL5

**DOI:** 10.1371/journal.pone.0089098

**Published:** 2014-02-18

**Authors:** Sharon A. Singh, Tracie A. Goldberg, Adrianna L. Henson, Sehba Husain-Krautter, Abdallah Nihrane, Lionel Blanc, Steven R. Ellis, Jeffrey M. Lipton, Johnson M. Liu

**Affiliations:** 1 Department of Molecular Medicine, Hofstra North Shore-LIJ School of Medicine, Hempstead, New York, United States of America; 2 The Feinstein Institute for Medical Research, Manhasset, New York, United States of America; 3 Division of Hematology/Oncology, Steven and Alexandra Cohen Children's Medical Center of New York, New Hyde Park, New York, United States of America; 4 Department of Biochemistry, University of Louisville, Louisville, Kentucky, United States of America; 5 Department of Pediatrics, Hofstra North Shore-LIJ School of Medicine, Hempstead, New York, United States of America; University of Washington, United States of America

## Abstract

Diamond Blackfan anemia (DBA) is a rare inherited bone marrow failure syndrome caused by ribosomal protein haploinsufficiency. DBA exhibits marked phenotypic variability, commonly presenting with erythroid hypoplasia, less consistently with non-erythroid features. The p53 pathway, activated by abortive ribosome assembly, is hypothesized to contribute to the erythroid failure of DBA. We studied murine embryonic stem (ES) cell lines harboring a gene trap mutation in a ribosomal protein gene, either *Rps19* or *Rpl5*. Both mutants exhibited ribosomal protein haploinsufficiency and polysome defects. *Rps19* mutant ES cells showed significant increase in p53 protein expression; however, there was no similar increase in the *Rpl5* mutant cells. Embryoid body formation was diminished in both mutants but nonspecifically rescued by knockdown of p53. When embryoid bodies were further differentiated to primitive erythroid colonies, both mutants exhibited a marked reduction in colony formation, which was again nonspecifically rescued by p53 inhibition. Cell cycle analyses were normal in *Rps19* mutant ES cells, but there was a significant delay in the G2/M phase in the *Rpl5* mutant cells, which was unaffected by p53 knockdown. Concordantly, *Rpl5* mutant ES cells had a more pronounced growth defect in liquid culture compared to the *Rps19* mutant cells. We conclude that the defects in our RPS19 and RPL5 haploinsufficient mouse ES cells are not adequately explained by p53 stabilization, as p53 knockdown appears to increase the growth and differentiation potential of both parental and mutant cells. Our studies demonstrate that gene trap mouse ES cells are useful tools to study the pathogenesis of DBA.

## Introduction

Diamond Blackfan anemia (DBA) is a rare inherited bone marrow failure syndrome [1], [2], characterized primarily by red blood cell hypoplasia but also associated with congenital anomalies, short stature, and cancer predisposition [3]. Atypical presentations are common, ranging from hydrops fetalis to non-anemic patients with macrocytosis [2]. Significant differences in phenotype are apparent among family members and unrelated individuals with the same mutation, suggesting considerable influence by modifying genes. Extensive studies have allowed classification of the majority of cases of DBA within the family of ribosomopathies [4], [5]. About 60–70% of the patients are heterozygotes for ribosomal protein (RP) gene mutations or deletions [6], resulting in either a state of haploinsufficiency for these ubiquitous proteins [7] or possibly a dominant negative mechanism caused by missense mutations [8]. The gene most commonly mutated in DBA is *RPS19*, found in 25% of patients. *RPL5* is mutated in about 9% of patients with DBA. The only genotype-phenotype correlation observed so far is the high prevalence of congenital abnormalities in patients with *RPL5* or *RPL11* mutations [9], [10]. A recent report has also identified a small subset of DBA patients with an X-linked mutation in erythroid transcription factor, GATA-1, which now links DBA to non-ribosomal protein genes [11]. Patients with this and other non-RP gene mutations expand both the genotype and phenotype of DBA, and the possibility that RP and non-RP gene mutations lead to similar molecular defects requires further study [12].

Although the molecular bases leading to the erythroid lineage specificity as well as other abnormalities in DBA remain largely unknown, it has been hypothesized to occur in part because the affected tissues are rapidly proliferating leading to a high demand for ribosomes [13]. Haploinsufficiency for ribosomal proteins is believed to lead to the failure of red cell production due to apoptosis [14], [15] and/or decreased proliferation due to cell cycle arrest of erythroid progenitors [16]. In addition, haploinsufficiency of ribosomal proteins decreases the efficiency of ribosome assembly triggering nucleolar stress [17] resulting in enhanced translation of other ribosomal protein mRNAs (5′-terminal oligopyrimidine tract [5′-TOP] containing mRNAs) [18]. Ribosomal proteins such as RPL11, RPL5, RPL23, RPS7 and RPS3 [19]–[22] have been previously suggested to bind to and inhibit the activity of an E3 ubiquitin ligase, HDM2 (MDM2 in mice) in contexts of nucleolar stress. HDM2 acts as the major regulator of steady state levels of p53 by maintaining low levels of p53 in normal, unstressed cells. In DBA, the inhibition of HDM2 by excessive free ribosomal proteins in this nucleolar stress pathway has been proposed to lead to an accumulation of p53 in cells, which could be the crux that links ribosomal gene mutations with apoptosis and cell cycle arrest. Animal models have indicated that p53 activation plays a key role in the disease pathophysiology and that p53 inhibition can lead to rescue of some or all of the disease manifestations [23].

We created cellular models of DBA using murine embryonic stem (ES) cells harboring gene trap mutations [24], [25] in *Rps19* or *Rpl5*. Murine ES cells, which have not been previously used as a disease model in DBA, are a powerful tool for the study of hematopoiesis and development in other tissues [26], [27]. We used these gene trap ES cells to successfully form chimeric mice indicating they are pluripotent cells able to differentiate into all tissues of the mouse. However, we were unable to obtain germline transmission possibly due to early embryonic lethality. Embryos analyzed as early as E6 did not show the presence of the gene trap vector. Thus, the focus of our studies turned to the *in vitro* characterization and differentiation of the mutant ES cells.

Protocols for the hematopoietic differentiation of mouse ES cells are well established and have been shown to faithfully recapitulate in vivo erythroid (primitive and definitive) differentiation in the mouse embryo [28]–[30]. During mammalian development there are three waves of erythropoiesis: (i) primitive erythropoiesis from the yolk sac (ii) a transient wave of definitive erythroid precursors from the yolk sac that seed the fetal liver and (iii) definitive erythroid progenitors derived from the hematopoietic stem cell, originating from the fetal liver during gestation and the bone marrow postnatally [27]. Primitive erythropoiesis is believed to be critical to the early postimplantation embryo.

To study the ontogeny of primitive erythropoiesis in our DBA models, we subjected the ES cells to *in vitro* differentiation conditions that stimulate primitive erythropoiesis. Both RPS19 and RPL5 haploinsufficient ES cells exhibited a similar failure of primitive erythropoiesis. By RNA interference, we demonstrated a nonspecific rescue of primitive erythropoiesis with p53 knockdown. The *Rpl5* mutant had a severe delay in the G2/M transition at the ES stage, while no such defect was found in the *Rps19* mutant model. There was no rescue of the cell cycle defect in the *Rpl5* mutant cells after knockdown of p53. Mouse ES cells haploinsufficient for RPL5 demonstrated an early p53-independent cell cycle defect and more severe growth impairment, which appears to distinguish RPL5 from RPS19 haploinsufficient ES cells.

## Methods

### Cells

The *Rps19* mutant murine ES cell line, YHC074, obtained from the Mutant Mouse Regional Resource Center, was created by electroporation of its parental cell line E14Tg2a.4 (mouse strain 129P2/OlaHsd) with the gene trap vector pGT0lxf, resulting in insertion of the vector within intron 3 of the *Rps19* gene. The *Rpl5* mutant murine ES cell line, D050B12, obtained from the German Gene Trap Consortium, was created by electroporation of parental cell line TBV-2 (mouse strain 129S2/SvPas) with the rFlipROSAbeta-Geo*+1 gene trap vector, leading to insertion of the vector within intron 3 of the *Rpl5* gene.

Cells were grown in ES maintenance media containing DMEM high glucose (Invitrogen), 15% fetal bovine serum ES-tested (FBS; StemCell Technologies), 0.1 mM non-essential amino acids (StemCell Technologies), 1% penicillin-streptomycin (Invitrogen), 2 mM L-glutamine (Invitrogen), 100µM monothioglycerol (MTG; Sigma) and 10 ng/mL mouse leukemia inhibitory factor (mLIF; StemCell Technologies). For growth curves, 5×10^3^ ES cells were seeded in 6 well-plates to provide enough wells for daily triplicate cell count for 5 days. Cells were trypsinized and counted using Trypan blue to exclude dead cells.

### Embryoid body formation

The cells were prepared for differentiation [31] (see Methods S1) and then plated (2×10^3^ cells/mL to 5×10^4^ cells/mL) in triplicate in low-adherence 35 mm Petri dishes (StemCell Technologies) with primary differentiation media (see Methods S1) to generate embryoid bodies (EBs). EBs were fed on day 7 with EB feed media composed of 50% primary differentiation media, supplemented with 15% FBS, 150µM MTG, 160 ng/mL recombinant mouse stem cell factor (rmSCF; StemCell Technologies), 30 ng/mL murine interleukin-3 (rmIL-3; StemCell Technologies), 20 ng/mL human interleukin-6 (rhIL-6; StemCell Technologies), 3 U/mL human erythropoietin (Epo; Amgen) and IMDM (StemCell Technologies). EBs were counted on day 4 for absolute numbers, then on day 10–12 for hematopoietic EB percentage in a blinded fashion. A hematopoietic EB was defined as an EB with erythroid and/or myeloid cells clustered at the edges. They are typically larger in size than non-hematopoietic EBs. EB counts were normalized to that of the respective parental cells (quantity of parental EBs set to 100).

### Primitive erythroid differentiation

Day 4–5 EBs were harvested, trypsinized, mechanically disrupted and added to primitive erythroid differentiation media containing basic methylcellulose, 15% plasma-derived fetal bovine serum (Animal Technologies), 2 mM L-glutamine, 0.45 mM MTG, 20% BIT 9500 (StemCell Technologies), 5 U/ml Epo, 50 µg/mL ascorbic acid, 5% Protein Free Hybridoma Medium-II (Invitrogen) and IMDM to achieve cell concentrations of 1×10^5^ cells/mL. Cells were plated in triplicate onto low-adherence 35 mm Petri dishes and primitive colonies were counted in a blinded fashion on day 7 of culture. Colony counts were normalized to that of the respective parental cells (quantity of parental colonies set to 100).

### Definitive erythroid differentiation

Day 7 EBs were harvested, incubated in Tryple E (Gibco), mechanically disrupted and added to definitive hematopoietic differentiation media containing basic methylcellulose, 15% FBS, 2 mM L-glutamine, 150µM MTG, 20% BIT9500 (StemCell Technologies), 150 ng/ml rmSCF, 30 ng/ml rmIL-3, 30 ng/ml rhIL-6, 3 U/ml Epo and IMDM to achieve a cell concentration of 1×10^5^ cells/mL. Cells were plated in triplicate onto low-adherence 35 mm Petri dishes. Definitive erythroid colonies (BFU-E and CFU-E) were counted on day 7 of culture in a blinded fashion.

### Stable transfection

The *Rpl5* mutant cell line D050B12 was transfected, using FuGene HD (Promega), with a pCMV6-A-Puro vector containing wild type Rpl5 cDNA and a puromycin resistance gene (Origene) to establish a stably transfected clone overexpressing Rpl5.

### Transient siRNA transfections

Twenty-four hours prior to primary differentiation, small interfering RNA (siRNA) targeting p53, (Dharmacon; see Methods S1) were transiently transfected into pre-differentiation cell cultures, using DharmaFect 1 (Dharmacon) transfection reagent according to the manufacturer's specifications. Non-targeting siRNAs (Dharmacon; Methods S1) were used as negative controls for the experiments.

### Antibodies

Mouse monoclonal antibody against RPS19 was from Abnova (Taiwan). Rabbit polyclonal antibody to RPL5 was from Abcam (Cambridge, MA). Rabbit polyclonal antibody, raised against a full-length human p53 fusion protein, was from Cell Signaling Technology (Danvers, MA). Mouse monoclonal antibody against β-Actin was from Santa Cruz Biotechnology (California). Goat anti-rabbit IgG horseradish peroxidase [HRP]-linked antibody was from Cell Signaling Technology. Goat anti-mouse IgG HRP-linked secondary antibody was from Santa Cruz.

### Western blot analysis

ES cells and EBs were lysed with protein lysis buffer containing 0.15 M sodium chloride (Kirkegaard & Perry labs/KPL), 1% Triton X-100 (KPL), 0.05 M Tris-HCl (KPL), 1% protease inhibitor cocktail (KPL) and distilled water, and 2.5 µg to 10 µg of total protein suspended in 4x Nupage loading buffer (Invitrogen) was boiled and loaded on 4–12% Bis-Tris Ready gels (Invitrogen). After transferring proteins to nitrocellulose membranes (Biorad), western blots were performed as described previously [32]. Immunoreactive bands were detected by the enhanced chemiluminescence method (Pierce Chemical). Relative quantification of western blot data was performed using the Image J software.

### Polysome profiles

ES cells, grown to 80% confluence, were incubated with 1% (vol/vol) of 9 mg/mL cycloheximide (Sigma Aldrich) for 10 minutes at 37°C and then trypsinized. Cells were washed with PBS and lysed at 4°C using a handheld homogenizer (Fisher Scientific) in polysome buffer containing 50 mM Tris-HCl (Fisher Scientific), 240 mM NaCl (Fisher Scientific), 10 mM MgCl_2_ (Sigma Aldrich), 5 mM beta-mercaptoethanol (Sigma Aldrich), 250 mM sucrose (Fisher Scientific), 2% Triton X (Sigma Aldrich), 100µg/mL heparin (Alfa Aesar), and 90µg/mL cycloheximide. Lysates were run on 15–55% sucrose gradients containing 25 mM Tris-HCl, 25 mM NaCl and 5 mM MgCl_2_. Gradients were centrifuged at 28,000 rpm for 7–8 hours using a Beckman L8-M ultracentrifuge. The gradients were then broken down using an ISCO density gradient fractionator, retriever and UA-6 UV/Vis detector (ISCO).

### Cell cycle analysis

ES cells, plated at equal density 24 hours prior to cell cycle analysis, were harvested, washed with PBS and fixed in chilled 70% ethanol for 1 hour. After fixation, cells were incubated with 0.5% RNase A and 4% Propidium Iodide (PI) (Sigma Aldrich) in PBS for 30 minutes at 37°C. Fluorescence intensity was measured using a FACSCalibur flow cytometer (Becton Dickinson). All analyses were performed using FlowJo software v9.2 (TreeStar).

### Quantitative real time RT-PCR

Total RNA was extracted from ES cells and hematopoietic colonies using the High Pure RNA isolation kit (Roche) according to manufacturer's protocol. First strand cDNA was generated from RNA with the Transcriptor First Strand cDNA synthesis kit, and quantitative real-time reverse-transcription PCR (qRT-PCR) was performed using the LightCycler 480instrument and kit (Roche) according to manufacturer's protocol. GAPDH and β-actin were used as internal controls. Relative changes of mRNA amounts were calculated using the ΔΔC_t_ method. All target gene primer-probe sets were designed by Roche (Methods S1).

### Statistics

Statistical significance of *in vitro* differentiation functional studies was evaluated using 2-tailed paired Student's t- test. (* p<0.05; ** p<0.01 for all figures). Error bars were generated using the standard error of the mean.

## Results

### *Rps19* and *Rpl5* mutant ES cells exhibit protein haploinsufficiency and ribosomal subunit assembly defects

**Figure 1:**
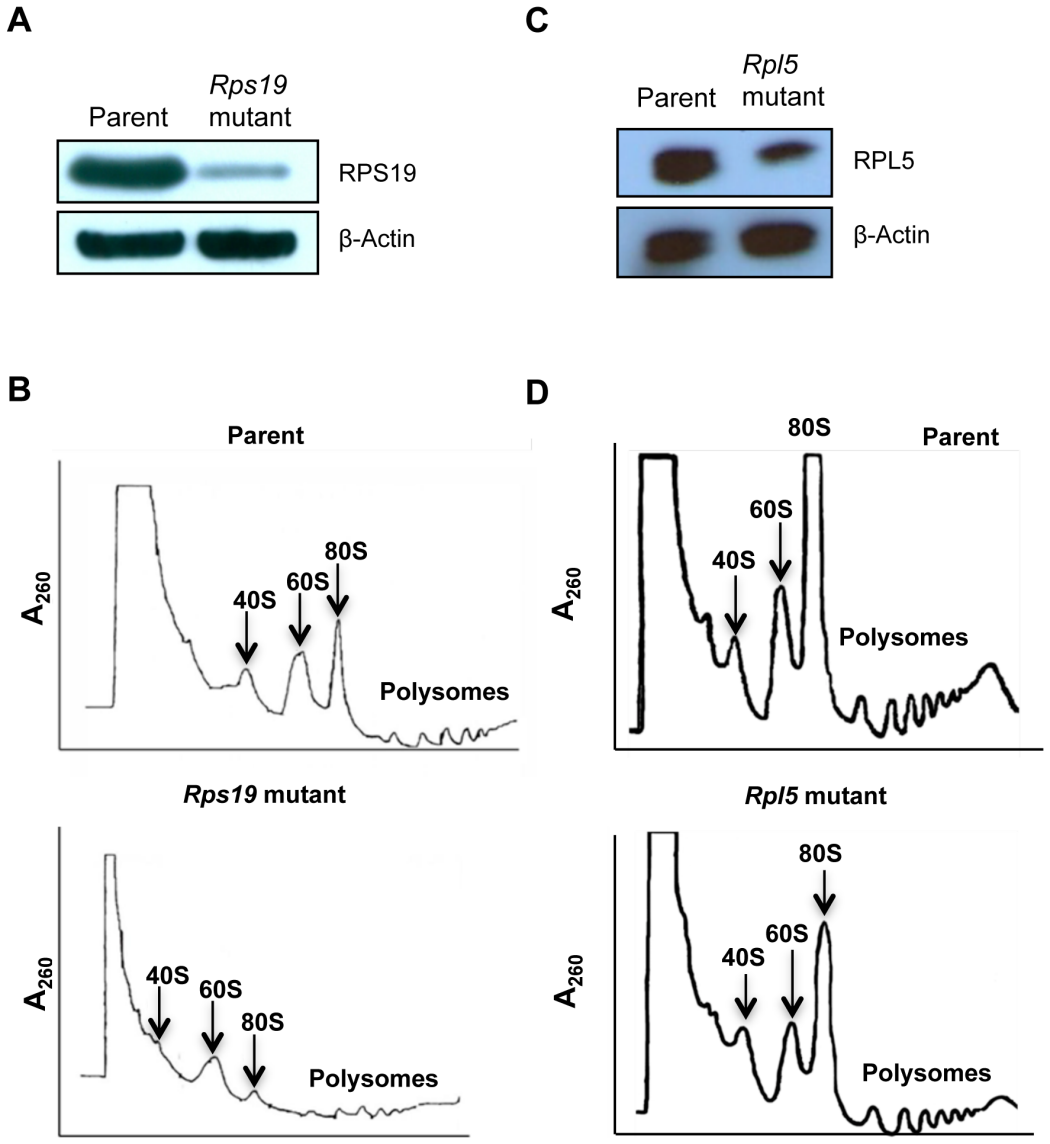
Protein haploinsufficiency and polysome defects in *Rps19* and *Rpl5* mutant mouse embryonic stem cells. For immunoblotting, ES cell lysates were separated using gel electrophoresis, transferred to a nitrocellulose membrane, and blotted with antibodies against RPS19 and RPL5. β-Actin was used as a loading control. *Rps19* mutant (A) and *Rpl5* mutant (C) ES cells showed protein haploinsufficiency (upper panels); β-Actin confirmed similar protein loading for mutant and parent (lower panels). For analyses of polysome profiles, ES cells were incubated in the presence of cycloheximide, lysed, and layered onto sucrose gradients. After ultra-centrifugation, polysome profiles were retrieved using an ISCO density gradient fractionator and UA-6 UV/Vis detector. RPS19 haploinsufficient ES cells (B, lower panel) showed a decreased 40S peak when compared to the parental line (B, top panel). In contrast, RPL5 haploinsufficient cells (D, lower panel) had a decreased 60S subunit compared with the parental cells (D, top panel).

In an attempt to confirm the effects of gene trapping on the mutant cells, expression levels of the *Rps19* mutant (YHC074, Figure 1A) and the *Rpl5* mutant (D050B12, Figure 1C) at the ES cell stage were assessed by western blot. We observed that both cells exhibited reduced amounts of their respective ribosomal proteins. In addition, *Rpl5* mutant ES cells were transfected with a plasmid containing Rpl5 wild type cDNA, and the efficiency of correction was evaluated by qRT-PCR. A clone that expressed roughly twice the amount of mRNA as the mutant was then selected (Figure S1) for rescue experiments.

Mutations in ribosomal proteins often lead to aberrant ribosome assembly. Therefore, to better characterize the mutant phenotypes, we analyzed the polysome profiles for both mutants. *Rps19* mutant ES cells demonstrated a markedly reduced 40S peak, a reduced 80S peak and decreased polysome peaks, compared to its parental cell line (Figure 1B). This pattern corresponds to a decrease in small ribosomal subunit assembly, consistent with the phenotype observed in cells carrying a mutation in a small ribosomal subunit protein gene. In contrast, the polysome profile of *Rpl5* mutant ES cells showed a reduced 60S peak and a reduction in polysomes compared to its parental cell line, which is expected with a deficiency of a large ribosomal subunit protein (Figure 1D).

### Both *Rps19* and *Rpl5* mutant cells exhibit decreased embryoid body formation

**Figure 2:**
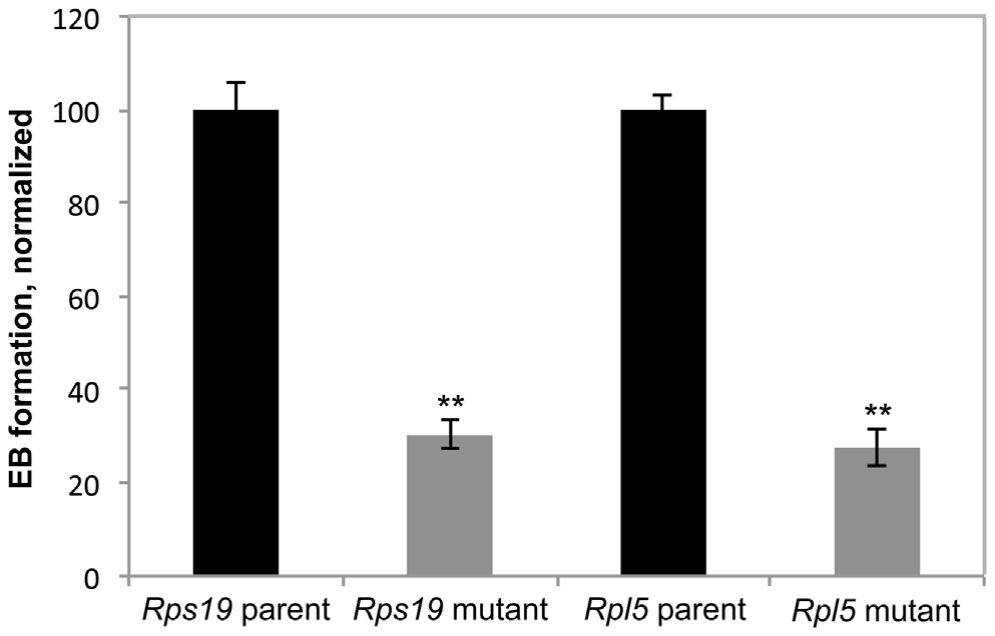
Embryoid body (EB) formation is impaired in both *Rps19* and *Rpl5* mutants. ES cells were differentiated into EBs and scored on day 4 to assess total number of EBs formed. Both mutants showed a reduction in EB formation when compared to the parental cells (3 independent experiments).

Functional defects intrinsic to these mutant cells were assessed by *in vitro* differentiation assays. Primary differentiation experiments were first used to measure the efficiency of embryoid body (EB) formation, defined as the number of EBs formed per ES cell plated, an *in vitro* representation of the differentiation potential of the mutant cells. The efficiency of embryoid body generation from ES cells was significantly reduced in both *Rps19* and *Rpl5* mutant cells (Figure 2).

### Both mutants exhibit a defect in primitive erythropoiesis

**Figure 3:**
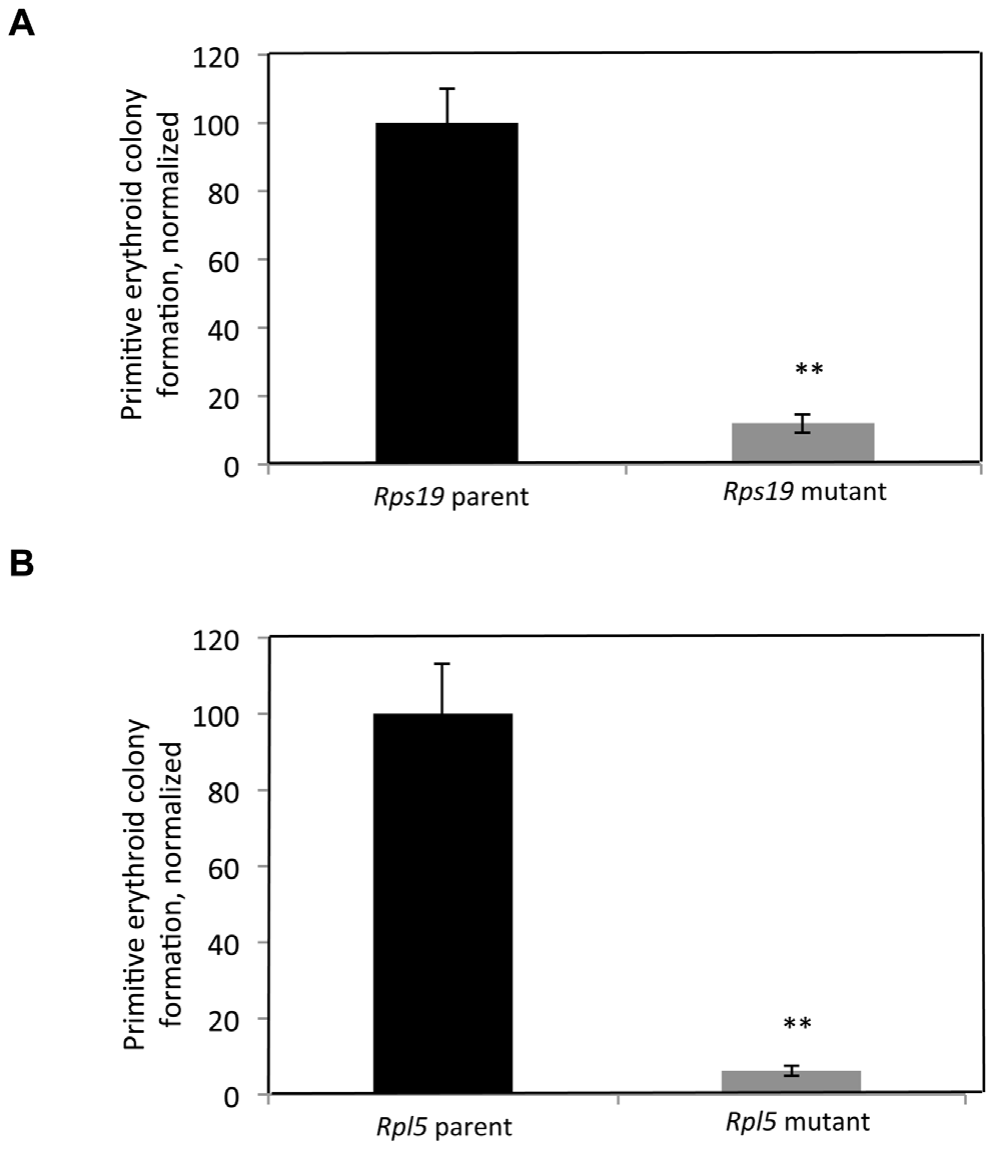
Primitive erythropoiesis is defective in *Rps19* and *Rpl5* mutants. Day 4–5 EBs were harvested, made into single cell suspension, and added to primitive erythroid differentiation media. Colonies were scored on day 7. Both *Rps19* mutant (A) and *Rpl5* mutant (B) cell lines exhibited a severe defect in primitive erythroid colony formation. (*Rpl5*-5 independent pooled experiments, *Rps19*-3 independent pooled experiments).

Primitive erythropoiesis assays, performed on day 4–5 EBs, and definitive erythropoiesis assays, performed on day 7–11 EBs, produced morphologically distinct erythroid colonies (Figure S2A). Hemoglobin qRT-PCR was used to confirm the identity of these colonies. As expected, primitive erythroid colonies showed a markedly higher ratio of mouse embryonic hemoglobin (Hbb-βh1) mRNA expression to mouse adult hemoglobin (Hbb-β1) mRNA expression, compared with definitive BFU-Es (Figure S2B). There were less definitive erythroid colonies formed in the *Rps19* and *Rpl5* mutants compared to the parental lines (Figure S3), consistent with the failure of definitive erythropoiesis in the majority of DBA patients. Primitive erythropoiesis, assessed by the total number of colonies formed, was markedly decreased in both the *Rps19* and *Rpl5* mutants (Figure 3A and B). The significance of this failure of primitive erythropoiesis is unclear, as the majority of DBA patients present postnatally.

### Differentiation defects in both *Rps19* and *Rpl5* mutants can be rescued non-specifically by p53 knockdown

**Figure 4:**
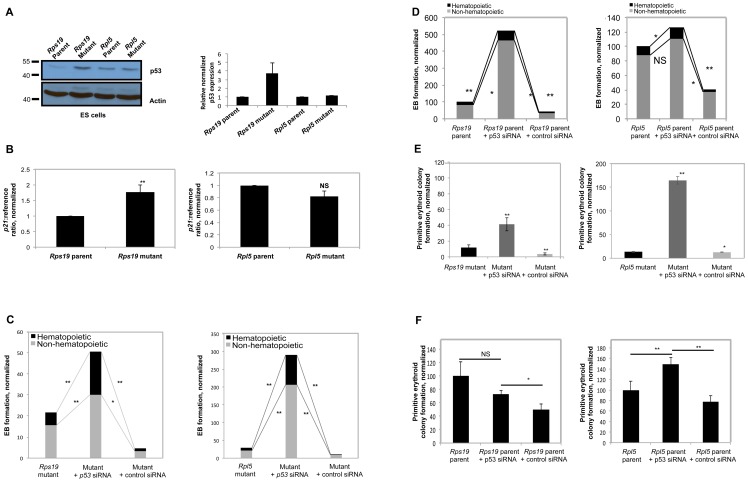
The differentiation defects observed in *Rps19* and *Rpl5* mutants are nonspecifically rescued by p53 inhibition. (A) Western blot analyses were performed from mutant ES cells with antibodies against p53, using β-Actin as a loading control. ES cells from the *Rps19* mutant cells showed an increase in p53 expression. In contrast, the *Rpl5* mutant expressed no increase in p53, compared with the parental line. Image J quantification of western blots from 3 independent experiments demonstrated that the *Rps19* mutant ES cells had approximately a 4-fold increase in p53 protein compared to the wild type cells. (B) qRT-PCR performed on these ES cells showed an increase in p21 mRNA only in the *Rps19* mutant ES cells (3 independent experiments) while there was no similar increase in the *Rpl5* mutant ES cells (4 independent experiments). siRNA targeting p53 was used to transiently transfect ES cells 24 hours prior to primary differentiation, obtaining >90% p53 knockdown by qRT-PCR. Both mutants (C) showed a significant increase in EB formation with p53 knockdown (4 independent pooled experiments). This effect was nonspecific, as p53 knockdown of parental cells also increased EB formation (D). The primitive erythroid colony defect was partially compensated in the *Rps19* mutant after p53 inhibition and overcompensated in the *Rpl5* mutant (E) (3 independent pooled experiments). This augmentation of colony formation was again nonspecific, as there was an increase in primitive colony formation with p53 knockdown in both parental ES cells when compared with the control siRNA (3 independent pooled experiments for *Rpl5* parent and 4 independent experiments for *Rps19* parent) (F).

p53 expression was evaluated at the protein level in both the *Rps19* and the *Rpl5* mutant ES and EB cells by western blot. As shown in Figure 4A (left panel), while a marked increase in p53 was observed in the *Rps19* mutant ES cells, no changes were found in the *Rpl5* mutant ES cells. This data was reproducible, as assessed by quantification of the western blots (Figure 4A, right panel), and similar results were obtained at the EB stage (Figure S4A). Note that basal expression levels for p53 differ in the two parental ES cells. This may be explained by differences in the mouse genetic background from which the ES cells have been produced [33]. In order to further validate the difference in p53 levels in these two mutants, p21, a downstream target of p53, was analyzed. *Rps19* mutant ES cells had a significant increase in p21 mRNA levels, while there was no change in p21 transcription in the *Rpl5* mutant (Figure 4B). This data was consistent with the results seen in the p53 western blot.

Transient p53 knockdown of both mutant ES cells was achieved by the addition of short interfering RNA (siRNA) to ES cells one day prior to EB generation. qRT-PCR performed 24 hours after addition of the siRNA resulted in >90% reduction in p53 mRNA transcription in all experiments (Figure S4B). Cells were subjected to primary differentiation, followed by secondary differentiation to primitive erythroid colonies. Knockdown of p53 in both mutants resulted in a significant increase in both non-hematopoietic and hematopoietic EB generation (Figure 4C). As a control, transfections using a non-targeting siRNA did not increase the EB formation efficiency in either mutant. However, knockdown of p53 in the parental lines resulted in a similar increase in EB formation, indicating that the effect of p53 knockdown on EB formation is not specific for the mutant cells (Figure 4D).

Transient p53 knockdown in the *Rps19* and *Rpl5* mutant cells led to a significant increase in primitive erythroid colony formation, whereas the control siRNA had no effect on colony formation (Figure 4E). Knockdown of p53 in *Rps19* mutant cells augmented primitive erythropoiesis to a lesser extent than the *Rpl5* mutant. In both of the wild type ES cells, p53 knockdown increased primitive colony formation relative to the effect of the control (non-targeting) siRNA, attesting to the lack of specificity of the p53 knockdown effect (Figure 4F). Of note, parental cells transfected with control siRNA had decreased colony formation when compared to non-transfected cells, implying inherent toxicity of the transfection process.

### *Rpl5* mutant shows p53 independent G2/M cell cycle delay at the ES stage

**Figure 5:**
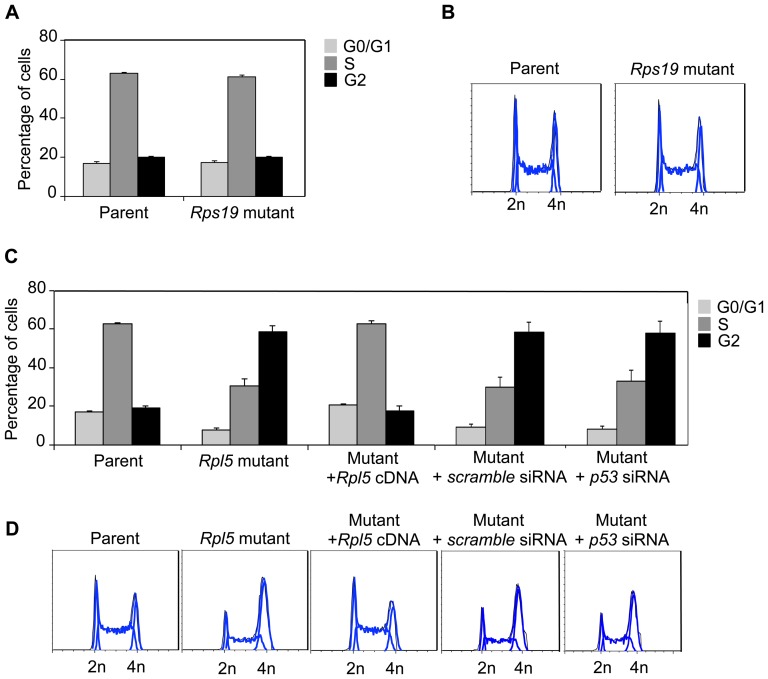
*Rpl5* mutant ES cells exhibit a p53-independent cell cycle arrest. Cell cycle analyses were performed by fixing ES cells with 70% ethanol, followed by staining with PI solution containing RNase A. Quantification of cell cycle phases (A), along with flow cytometry profiles (B) of *Rps19* mutant ES cells show no difference, compared to the parent. In contrast, the cell cycle profile of the *Rpl5* mutant ES cells exhibited a three-fold increase in the G2 phase with a concomitant decrease in the G1 and S phases, consistent with a delayed G2 phase transition (A, C) (three independent pooled experiments). Stable transfection of the *Rpl5* mutant with a vector containing Rpl5 cDNA showed complete correction of the cell cycle defect; however, siRNA knockdown of p53 was unable to rescue the defect (D).

Cell cycle analyses at the ES cell stage showed a difference between the *Rps19* mutant and the *Rpl5* mutant. The cell cycle status of the *Rps19* mutant was essentially unchanged, compared to its parent cells (Figure 5A &B). On the other hand, the *Rpl5* mutant exhibited a significant increase in the percentage of cells in the G_2_/M phase with a concomitant decrease of cells in the G1 and S phases, consistent with a G_2_ cell cycle delay (Figure 5C &D). The delay observed in the cell cycle was rescued by transfection of the mutant with wild type Rpl5 cDNA. To get further insights into the putative involvement of p53 in the observed cell cycle defect, p53 was knocked down in the *Rpl5* mutant. However, despite the high efficiency of the knockdown (97%), as evaluated by qRT-PCR, no difference was observed in the cell cycle. This data strongly suggests that the G2 phase defect observed is due to a mechanism independent of p53.

### *Rpl5* mutant ES cells grow more slowly compared to parental and *Rps19* mutant cells

**Figure 6:**
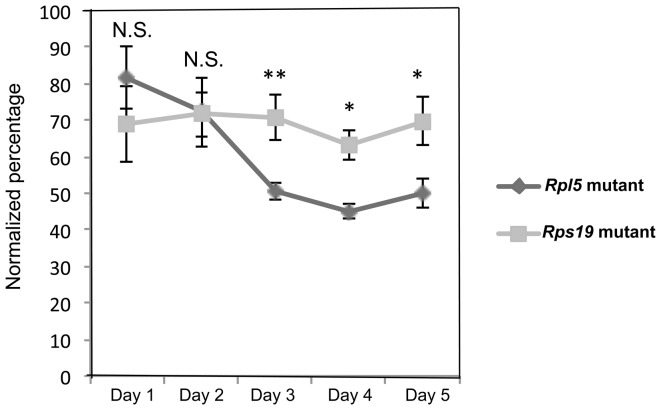
*Rpl5* mutant ES cells exhibit more severe growth defect than *Rps19* mutant cells. Cells were seeded in 6 well plates at a concentration of 5×10^3^ per well with ES maintenance media, and live cell counts were performed daily for 5 days using Trypan blue. The total number of cells from the two mutants were normalized to their respective parental line and represented as a percentage. From days 3–5 of culture, the *Rpl5* mutant ES cells expanded at a significantly slower rate, when compared with the *Rps19* mutant ES cells (three independent pooled experiments).

Both mutants showed a growth defect starting at day 3 of culture when compared to their respective parental cells (Figure S5). Mutant cell counts were normalized to their parental cell counts and represented as a percentage. The *Rpl5* mutant ES cells had a more severe growth defect when compared to the *Rps19* mutant cells from days 3–5 of culture (Figure 6). This difference may correlate with the early cell cycle abnormality seen in the *Rpl5* mutant ES cells.

## Discussion

DBA is a heterogeneous disorder that can manifest prenatally with congenital anomalies and hydrops fetalis, or postnatally with a failure of definitive erythropoiesis. In the present study, we developed a disease model using mouse embryonic stem cells. This model has the potential to elucidate the specific mechanisms underlying divergent DBA phenotypes, which can manifest at any point during development. Both primitive (yolk sac) and definitive erythroid colonies can be easily and efficiently generated and studied in parallel from the same differentiation culture. In addition, this ES differentiation system can be used to ascertain the response to potential experimental therapies (drugs or gene therapy) aimed at modifying the disease phenotype at specific stages of development. Robust gene knockdown with RNA interference can be achieved in ES cells, as demonstrated by this work. ES cells with gene trap mutations in other ribosomal proteins are readily available and can be used to further analyze correlations between genotype and phenotype. This report is the first to use such technology in DBA.

We chose to study mouse ES cells with gene trap mutations in *Rpl5* as well as *Rps19*, the most common gene mutated in DBA. By demonstrating protein haploinsufficiency, ribosome assembly defects and definitive erythroid differentiation defects, we have shown that our gene trap mutant mouse embryonic stem cell models faithfully recapitulate the major features of DBA. In our cellular model of DBA, both the *Rps19* and *Rpl5* mutants exhibit a severe defect in primitive erythropoiesis, which is in accord with others' findings in zebrafish and induced pluripotent stem cells [34]–[36]. We have expanded this finding to directly compare and contrast primitive and definitive erythropoiesis in quantifiable assays, which is a limitation in many other disease models. In the *Rps19* mutant ES cells, the defect in primitive erythropoiesis actually appeared more severe than in definitive erythropoiesis, suggesting the possibility of early embryonic loss in DBA.

**Figure 7:**
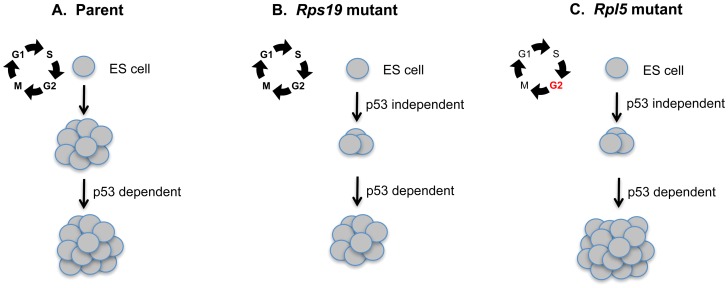
Proposed model suggesting a secondary role for p53 in augmenting erythroid colony formation in mouse ES cell models of Diamond Blackfan anemia. Wild type mouse embryonic stem (ES) cells can be differentiated into primitive erythroid colonies (A). In the normal setting, colony formation can be further increased by p53 knockdown. (B) *Rps19* mutant ES cells exhibit defective primitive erythroid colony formation through an unknown p53-independent mechanism. However colony formation can be augmented by p53 knockdown through a separate p53 dependent pathway. (C) The *Rpl5* mutant ES cells show an early cell cycle defect at the ES cell stage that is p53-independent. These cells also exhibit a similar defect in primitive erythroid colony formation through a p53- independent mechanism. p53 knockdown in these cells increases colony formation to a greater degree than the *Rps19* mutant cells, for unknown reasons.

Many ribosomal haploinsufficient animal models have demonstrated that p53 knockdown can ameliorate their respective erythroid and/or morphological phenotypes [37]–[40]. A lingering question raised by these models is the specific role of p53 in the ribosomal protein haploinsufficient cell. In our model, p53 inhibition increased EB formation and primitive erythropoiesis in both the wild type and mutant ES cells, indicating that the role of p53 in growth and differentiation is largely nonspecific. This may reflect a general growth advantage seen in cells after p53 knockdown [41]. Possibly, this parallels the pro-survival effects of glucocorticoid steroids, which are used clinically to stimulate erythropoiesis in DBA patients [42], [43]. Glucocorticoids and other steroid hormones are well known stimulators of erythropoiesis in stress conditions, and pharmacologic doses of glucocorticoids can induce long-term proliferation of normal erythroblasts from a number of different species. We propose that the intrinsic differentiation defects caused by ribosomal protein haploinsufficiency are p53 independent, as there does not seem to be a direct correlation between basal p53 protein levels and the increase in colony formation after p53 knockdown. However, secondary pathways appear to augment erythropoiesis in both normal and haploinsufficient states in response to p53 knockdown, which can compensate for the intrinsic defect in haploinsufficient cells (see model Figure 7). For unknown reasons, this p53-dependent augmentation is greater in the setting of ribosomal protein haploinsufficiency. The mechanism underlying this finding requires further study.

Based on cell cycle analyses, we found that *Rpl5* mutant ES cells exhibited a delay in the G2/M cell cycle transition that was independent of p53 activation. Previous work by other investigators demonstrated a G0/G1 arrest in *Rps19* mutant hematopoietic progenitors [16], [44]. Studies in *Rps19* mutant fibroblasts showed a similar arrest, whereas *Rps24* mutants exhibited altered S phase and decreased G2/M changes [45]. A recent report also describes that disruption of both the 40S and 60S subunits leads to both a G1 and a G2/M arrest [46]. It is unclear why different ribosomal protein defects lead to different cell cycle abnormalities. Embryonic stem cells (both murine and human) have a capacity for unlimited proliferation while retaining totipotency, and are believed to exhibit a short G1 phase and a high proportion of cells in S phase [47]. Consequently, the specific G2/M defect seen in our *Rpl5* mutant is particularly striking, and we are actively engaged in determining the underlying mechanisms. Preliminary experiments using microarrays and qRT-PCR (data not shown) have uncovered significant differences in expression of certain cell cycle genes in the *Rpl5* ES cells when compared with parental controls.

Overall, our data is generally consistent with a recent publication from Teng et al. on depletion of RPL5/RPL11 in human lung fibroblasts [48]. In agreement with their findings, we did not observe induction of p53 in our *Rpl5* mutant ES cells. We also observed a significant delay in the progression through the cell cycle, with consequent impaired growth rate. However, we found an increased proportion of ES cells delayed at the G2/M phase, whereas Teng et al. did not in their fibroblasts. This may be due to intrinsic differences in the cell cycle between undifferentiated totipotent ES cells and differentiated lung fibroblasts. It will be important to determine if diminished translational capacity and suppressed cyclin production account for the cell cycle abnormality in our *Rpl5* mutant ES cells, as Teng et al. demonstrated in RPL5-depleted lung fibroblasts.

In this work, we have demonstrated that the *Rps19* and *Rpl5* mutant gene trap mouse embryonic stem cell models are useful tools to study the ontogeny of erythropoiesis and the pathophysiology of DBA. These two mutant ES cells exhibited similar defective EB formation and patterns of primitive and definitive erythropoiesis. Despite having similar differentiation defects, only *Rps19* mutant ES cells were found to have increased basal levels of p53. Knockdown of p53 provided a nonspecific growth and differentiation advantage to both normal and mutant ES cells. Furthermore, the *Rpl5* mutant ES cells exhibited a p53-independent G2/M cell cycle defect. We conclude that the growth and differentiation defects seen in ribosomal protein haploinsufficient ES cells may not be due to p53 stabilization via inhibition of MDM2.

## Supporting Information

Figure S1 ***Rpl5*** **mutant ES cells corrected by stable transfection with cDNA-containing vector.** FuGene^®^ was used to transfect the *Rpl5* mutant line with a vector containing *Rpl5* cDNA and a puromycin resistance gene (Origene). Transfected cells were grown in puromycin; resistant clones were selected and expanded. Total RNA was isolated, cDNA was synthesized, and qRT-PCR was performed for *Rpl5* expression, with *β-actin* and *Gapdh* used as reference genes to normalize the data. A clone was selected which showed increased levels of Rpl5 mRNA.
(TIF)

Figure S2 **Primitive erythroid colonies show expression of mouse embryonic hemoglobin (Hbb-βH1).** After isolation of total RNA from primitive (A, upper panel) and definitive (A, lower panel) erythroid colonies, qRT-PCR was performed to assess the expression levels of Hbb-βH1. Results were normalized with Gapdh and β-actin. The ratio of mouse embryonic hemoglobin (Hbb-βH1) to the major adult mouse hemoglobin (Hbb-β1) is shown (B). Primitive erythroid colonies showed high expression of embryonic hemoglobin, while the definitive erythroid colonies showed no expression. Scale bar represents 100µm.
(TIF)

Figure S3 ***Rps19*** **and** ***Rpl5*** **mutant ES cells form less definitive erythroid colonies** ***in vitro*** **.** Day 7 embryoid bodies were made into a single cell suspension, and 1×10^5^ cells were plated in methylcellulose media containing FBS, L-glutamine, monothioglycerol, BIT9500 (StemCell Technologies), Stem cell factor, IL-3, IL-6, 3 U/ml Epo and IMDM. Definitive erythroid colonies (BFU-E and CFU-E) were scored on day 7 in a blinded fashion. Fewer erythroid colonies were produced in the *Rps19* (A) and *Rpl5* (B) mutants, compared to the parent (three independent pooled experiments plated in triplicate).
(TIF)

Figure S4 **p53 quantification.** (A) Western blot on EB cells demonstrated an increase in p53 in the *Rps19* mutant but no increase in the *Rpl5* mutant EB cells. (B&C) p53 knockdown of *Rps19* and *Rpl5* mutants using RNA interference. Pooled siRNA targeting *p53* was used to transiently transfect mutant ES cells. Total RNA was isolated, cDNA was synthesized and qRT-PCR was performed with either β-actin or Gapdh to normalize *p53* expression. Over 90% knockdown of *p53* was achieved in all experiments in the *Rps19* (B) and *Rpl5* (C) mutant ES cells.
(TIF)

Figure S5 ***Rpl5*** **and** ***Rps19*** **mutant ES cells exhibit growth defects.** Cells were seeded in 6 well plates in ES maintenance media at a concentration of 5×10^3^ per well. Live cell counts were performed daily for 5 days using Trypan blue. Both mutants exhibited poor expansion in culture from days 3–5 (three independent pooled experiments in triplicate for each cell type).
(TIF)

Methods S1 (DOCX)
