# Correction: p53-Independent Cell Cycle and Erythroid Differentiation Defects in Murine Embryonic Stem Cells Haploinsufficient for Diamond Blackfan Anemia-Proteins: RPS19 versus RPL5

**DOI:** 10.1371/journal.pone.0096689

**Published:** 2014-04-30

**Authors:** The PLOS ONE Staff

## Notice of Republication

This article was republished on April 10, 2014, to correct formatting errors that were introduced during the typesetting process. The publisher apologizes for the errors. Please download this article again to view the correct version. The originally published, uncorrected article and the republished, corrected article are provided here for reference.

## Supporting Information

File S1 Originally published, uncorrected article.
(PDF)

File S2 Republished, corrected article.
(PDF)
