# Supplementary figures and images for: p53-Independent Cell Cycle and Erythroid Differentiation Defects in Murine Embryonic Stem Cells Haploinsufficient for Diamond Blackfan Anemia-Proteins: RPS19 versus RPL5
Source: PLoS One. 2014 Feb 18;9(2):e89098. doi: 10.1371/journal.pone.0089098 (PMC3928369; doi:10.1371/journal.pone.0089098)

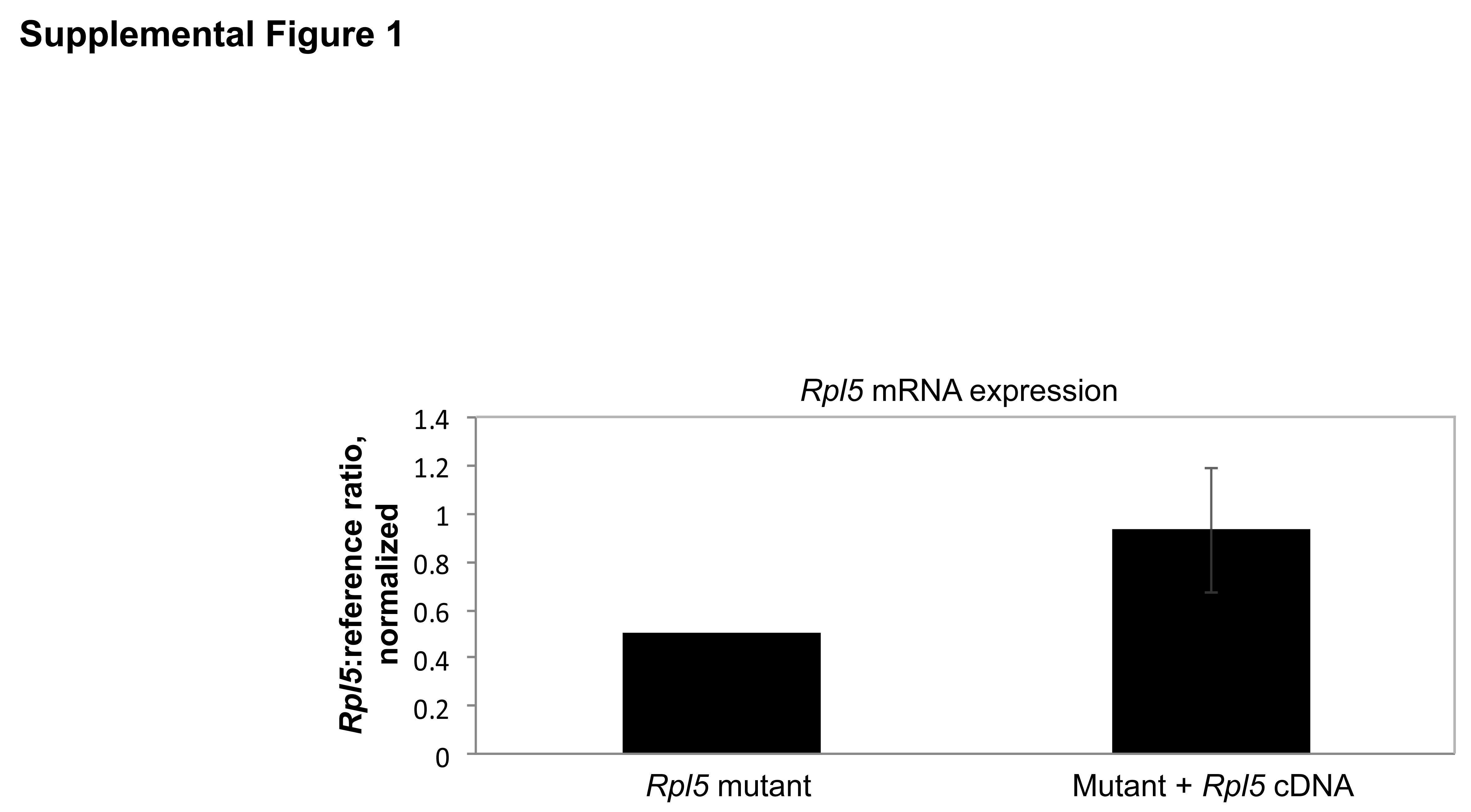

Supplement: Figure S1 — Rpl5 mutant ES cells corrected by stable transfection with cDNA-containing vector. FuGene® was used to transfect the Rpl5 mutant line with a vector containing Rpl5 cDNA and a puromycin resistance gene (Origene). Transfected cells were grown in puromycin; resistant clones were selected and expanded. Total RNA was isolated, cDNA was synthesized, and qRT-PCR was performed for Rpl5 expression, with β-actin and Gapdh used as reference genes to normalize the data. A clone was selected which showed increased levels of Rpl5 mRNA. (TIF) [file pone.0089098.s001.tif]

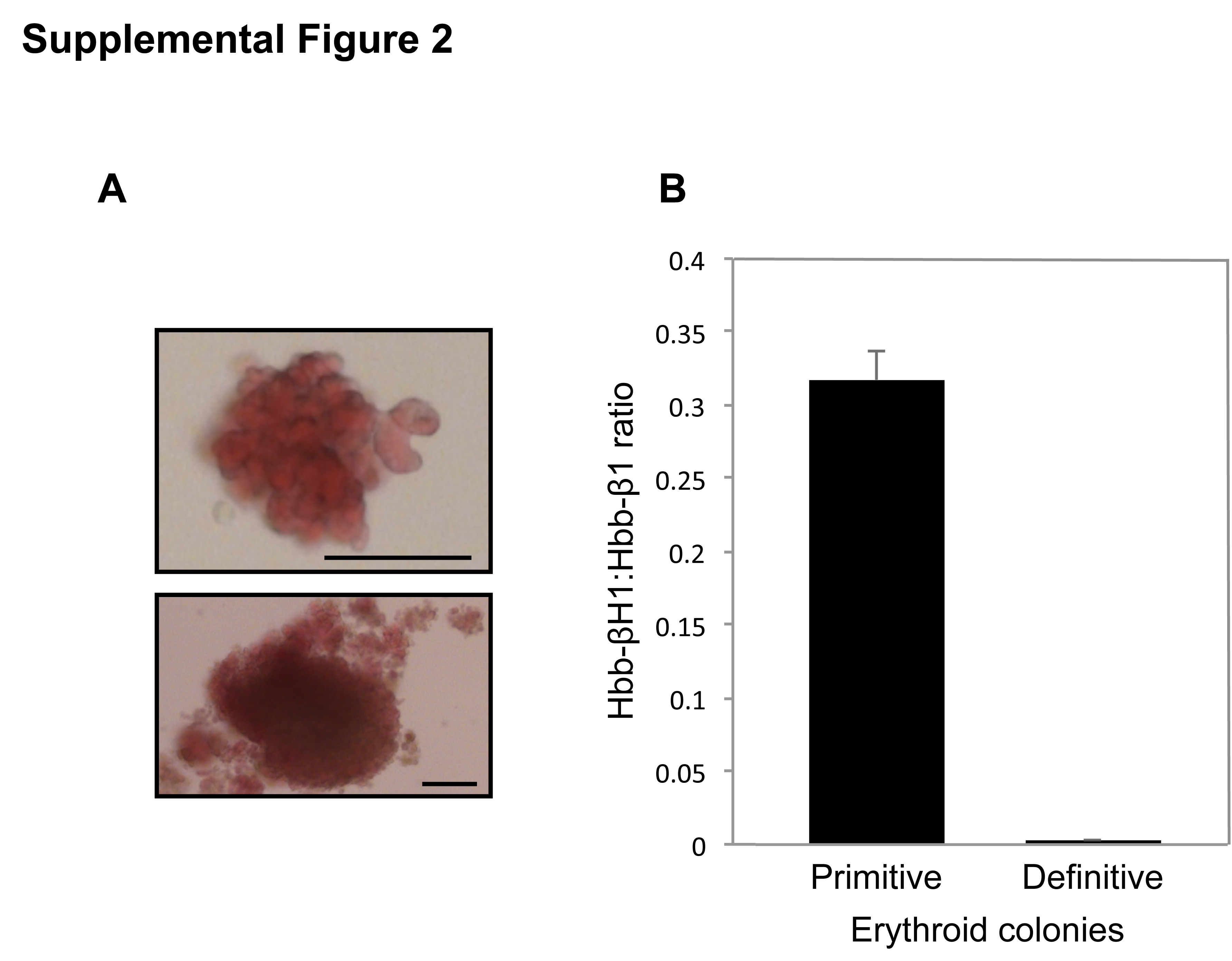

Supplement: Figure S2 — Primitive erythroid colonies show expression of mouse embryonic hemoglobin (Hbb-βH1). After isolation of total RNA from primitive (A, upper panel) and definitive (A, lower panel) erythroid colonies, qRT-PCR was performed to assess the expression levels of Hbb-βH1. Results were normalized with Gapdh and β-actin. The ratio of mouse embryonic hemoglobin (Hbb-βH1) to the major adult mouse hemoglobin (Hbb-β1) is shown (B). Primitive erythroid colonies showed high expression of embryonic hemoglobin, while the definitive erythroid colonies showed no expression. Scale bar represents 100µm. (TIF) [file pone.0089098.s002.tif]

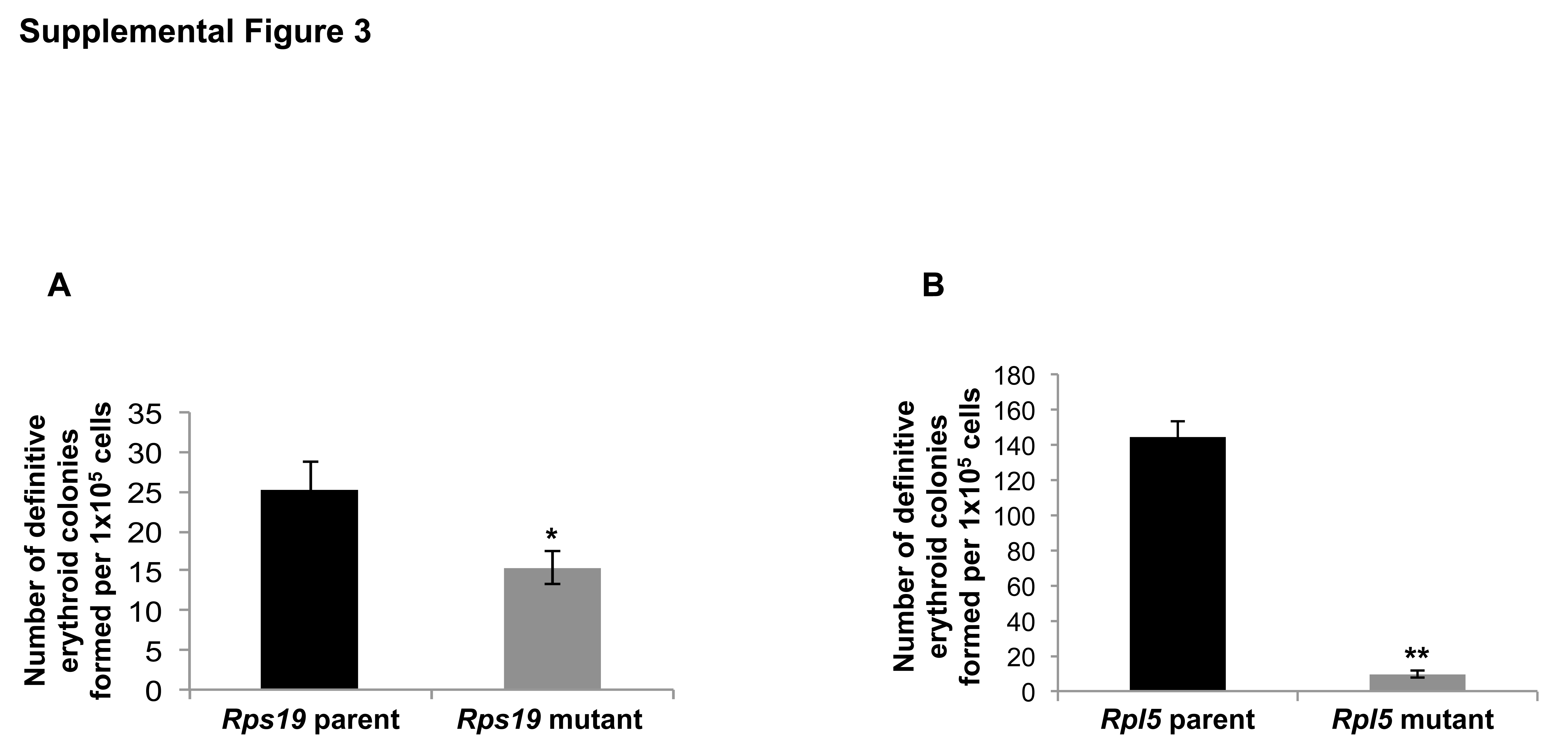

Supplement: Figure S3 — Rps19 and Rpl5 mutant ES cells form less definitive erythroid colonies in vitro . Day 7 embryoid bodies were made into a single cell suspension, and 1×10^5^ cells were plated in methylcellulose media containing FBS, L-glutamine, monothioglycerol, BIT9500 (StemCell Technologies), Stem cell factor, IL-3, IL-6, 3 U/ml Epo and IMDM. Definitive erythroid colonies (BFU-E and CFU-E) were scored on day 7 in a blinded fashion. Fewer erythroid colonies were produced in the Rps19 (A) and Rpl5 (B) mutants, compared to the parent (three independent pooled experiments plated in triplicate). (TIF) [file pone.0089098.s003.tif]

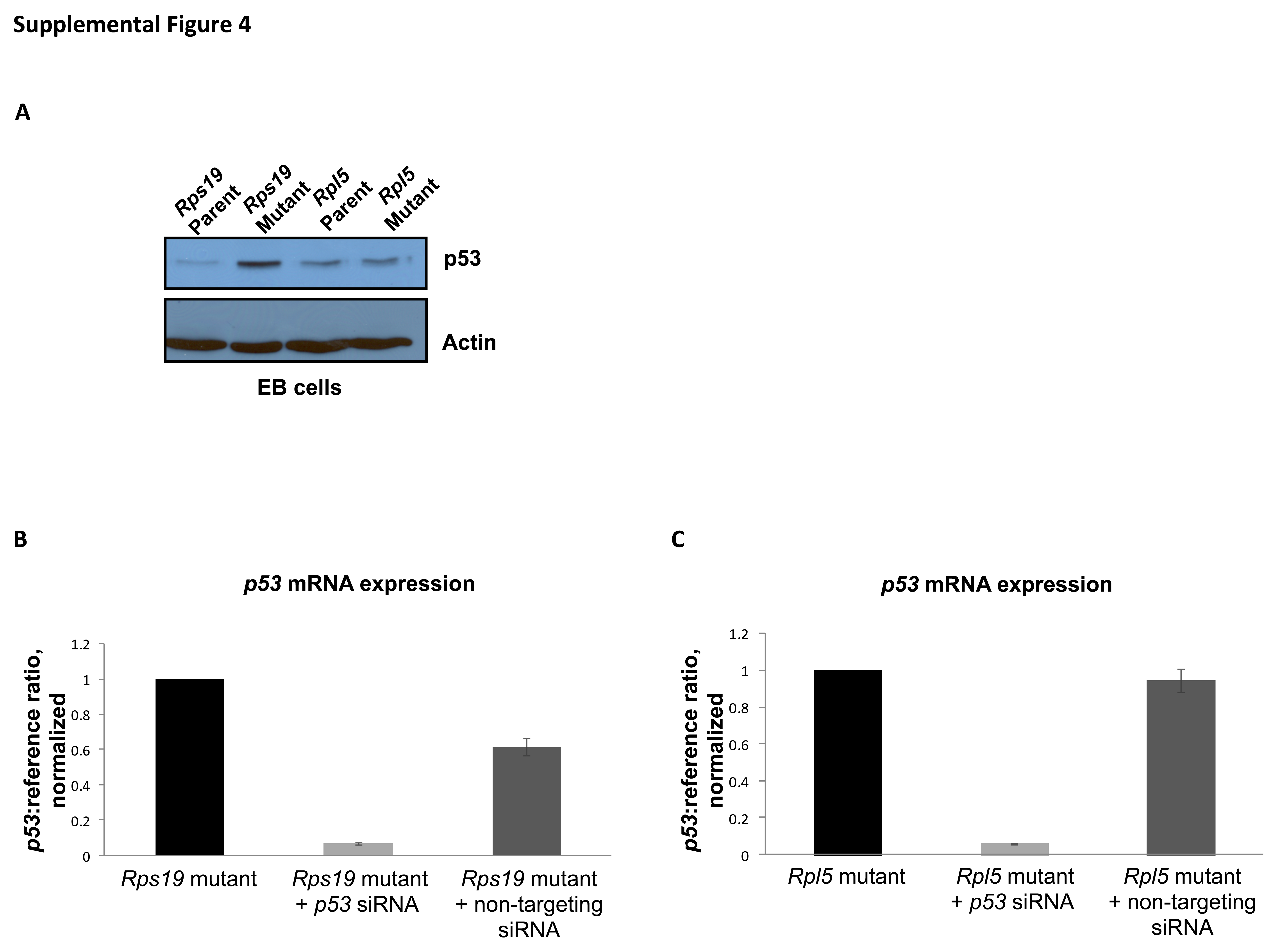

Supplement: Figure S4 — p53 quantification. (A) Western blot on EB cells demonstrated an increase in p53 in the Rps19 mutant but no increase in the Rpl5 mutant EB cells. (B&C) p53 knockdown of Rps19 and Rpl5 mutants using RNA interference. Pooled siRNA targeting p53 was used to transiently transfect mutant ES cells. Total RNA was isolated, cDNA was synthesized and qRT-PCR was performed with either β-actin or Gapdh to normalize p53 expression. Over 90% knockdown of p53 was achieved in all experiments in the Rps19 (B) and Rpl5 (C) mutant ES cells. (TIF) [file pone.0089098.s004.tif]

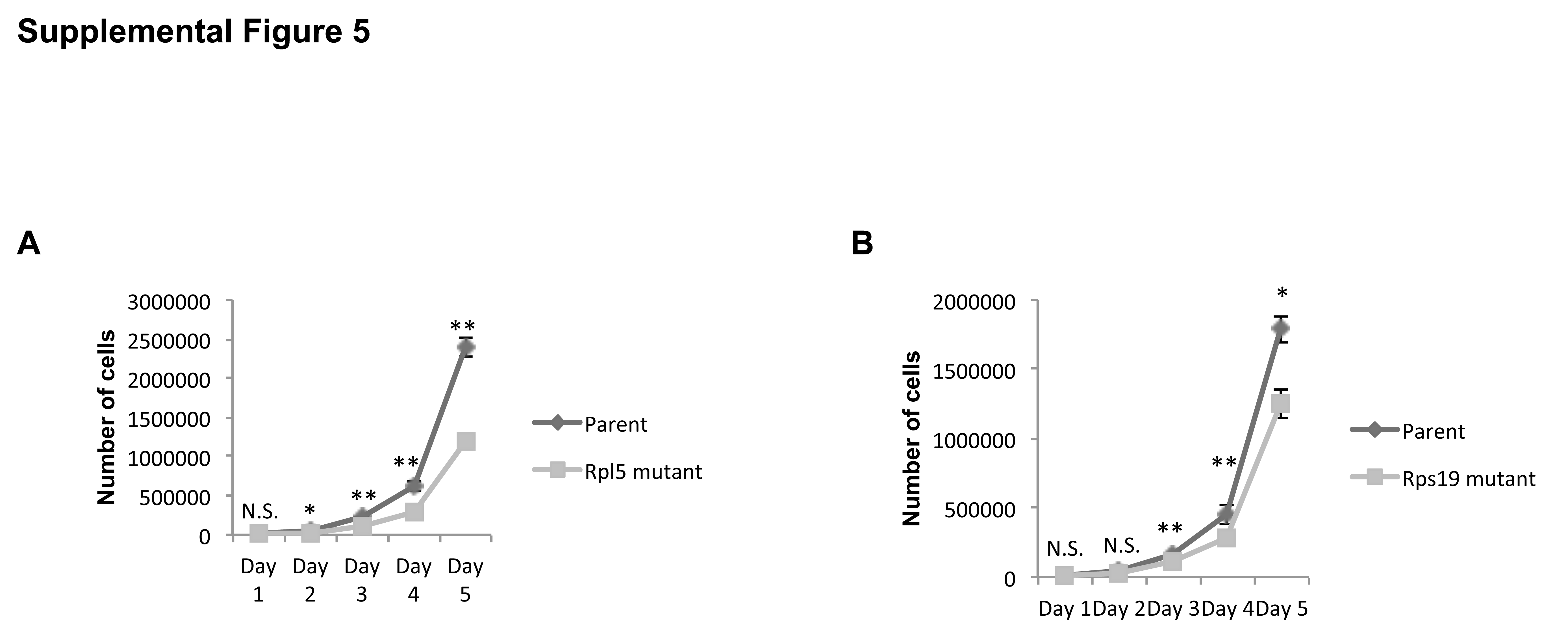

Supplement: Figure S5 — Rpl5 and Rps19 mutant ES cells exhibit growth defects. Cells were seeded in 6 well plates in ES maintenance media at a concentration of 5×10^3^ per well. Live cell counts were performed daily for 5 days using Trypan blue. Both mutants exhibited poor expansion in culture from days 3–5 (three independent pooled experiments in triplicate for each cell type). (TIF) [file pone.0089098.s005.tif]
