# Supplementary material for: p53-Independent Cell Cycle and Erythroid Differentiation Defects in Murine Embryonic Stem Cells Haploinsufficient for Diamond Blackfan Anemia-Proteins: RPS19 versus RPL5
Source: PLoS One. 2014 Feb 18;9(2):e89098. doi: 10.1371/journal.pone.0089098 (PMC3928369; doi:10.1371/journal.pone.0089098)
Supplement: Methods S1 — (DOCX) [file pone.0089098.s006.docx]

**Supplemental methods:**

**Cell culture**

YHC074 and E14Tg2a.4, both feeder-independent cell lines, were thawed and passaged onto gelatin-coated flasks (gelatin solution from StemCell Technologies) in the absence of fibroblasts. D050B12 and TBV-2, initially feeder-dependent cell lines, were thawed onto 10µg/mL mitomycin C (Sigma)-treated primary embryonic fibroblasts (StemCell Technologies) and subsequently plated onto gelatin-coated flasks without fibroblasts to establish feeder-free cells. Mutant cells were grown in Geneticin (Invitrogen) selection prior to differentiation. Two days prior to differentiation, cells were passaged into ES pre-differentiation media consisting of IMDM (StemCell Technologies), 15% FBS ES-tested, 2 mM L-glutamine, 1% penicillin-streptomycin, 0.1mM non-essential amino acids, 100μM MTG, and 10ng/mL mLIF. After 48 hours of incubation, cells were trypsinized with 1ml TrypLE Express (Gibco) and plated into primary differentiation media containing basic methylcellulose (StemCell Technologies), 15% FBS, 2mM L-glutamine, 150μM MTG, 40ng/mL murine stem cell factor (rmSCF; StemCell Technologies) and IMDM.

**qRT-PCR primer-probe sets designed by Roche**

([www.universalprobelibrary.com](http://www.universalprobelibrary.com)):

Hbb-b1: F primer TGCATGTGGATCCTGAGAAC; R primer GTGAAATCCTTGCCCAGGT; probe #92 (Roche)

Hbb-bh1: F primer TGGATCCTGAGAACTTCAAGC; R primer CATTGGCCACTCCAATCAC; probe #27 (Roche)

Rpl5: F primer GGTCTTGCCCGAACTACAAC; R primer AACCAGGGAATCGTTTGGTA; probe #58 (Roche)

p53: F primer ATGCCCATGCTACAGAGGAG; R primer AGACTGGCCCTTCTTGGTCT; probe #78 (Roche)

β-actin: F primer CTAAGGCCAACCGTGAAAAG; R primer ACCAGAGGCATACAGGGACA ; probe #64 (Roche)

GAPDH: F primer TGTGTCCGTCGTGGATCTGA; R primer CCTGCTTCACCACCTTCTTGA; probe CCGCCTGGAGAAACCTGCCA

P21 : F primer tccacagcgatatccagaca ; R primer ggacatcaccaggattggac; probe# 21(Roche)

**Dharmacon siRNA sequences used in this study**

Mouse TRP53, siGENOME SMARTpool (M-040642-02):

GAAUGAGGCCUUAGAGUUA; UGAAUGAGGCCUUAGAGUU; GAAGAUAUCCUGCCAUCAC; CGUAAACGCUUCGAGAUGU

siGENOME Non-Targeting siRNA #2 (D-001210-02-05):

UAAGGCUAUGAAGAGAUAC
